# Posterior Reversible Encephalopathy Syndrome in the Emergency Department: Case Series and Literature Review

**DOI:** 10.5811/westjem.2014.12.24126

**Published:** 2015-01-05

**Authors:** Ryan J. Thompson, Brian Sharp, Jeffery Pothof, Azita Hamedani

**Affiliations:** University of Wisconsin, Department of Emergency Medicine, Madison, Wisconsin

## Abstract

**Introduction:**

Posterior Reversible Encephalopathy Syndrome (PRES) often has variable presentations and causes, with common radiographic features—namely posterior white matter changes on magnetic resonance (MRI). As MRI becomes a more frequently utilized imaging modality in the Emergency Department, PRES will become an entity that the Emergency Physician must be aware of and be able to diagnose.

**Case Report:**

We report three cases of PRES, all of which presented to the emergency department of a single academic medical center over a short period of time, including a 53-year-old woman with only relative hypertension, a 69-year-old woman who ultimately died, and a 46-year-old woman who had a subsequent intraparenchymal hemorrhage.

**Conclusion:**

PRES is likely much more common than previously thought and is a diagnosis that should be considered in a wide variety of emergency department patient presentations.

## INTRODUCTION

Posterior Reversible Encephalopathy Syndrome (PRES) was first described in 1996 by Hinchey et al*.* as Reversible Posterior Leukoencephalopathy Syndrome.^1^ PRES is characterized by a constellation of symptoms including visual changes, headache, altered mental status, and seizures and is associated with white matter edema in the posterior parietal-temporal-occipital regions, most often visualized on magnetic resonance imaging (MRI).

As the name implies, patients with PRES typically have resolution of symptoms within days of the initiation of treatment of the underlying cause, although MRI findings can take weeks to fully resolve.^2^ There are however case reports of patients experiencing brain herniation^3^ and death^4^ secondary to PRES.

With the increase in advanced MRI in the Emergency Department (ED), it is likely that Emergency Physicians will be able to effectively diagnose PRES and should consider this as part of the working differential diagnosis of a variety of key chief complaints in the ED. Awareness of the range of patient presentations and outcomes will allow optimal management of such patients earlier in their presenting course. We present three patients with variable presentations who presented in a very short period of time to our emergency department with PRES.

## CASE SERIES

### Case 1

A previously healthy 53-year-old woman, with a past medical history notable only for mild esophageal dysmotility, presented to a local Urgent Care with several hours of nausea, vomiting, and diarrhea. She was treated with 4mg intravenous (IV) ondansetron and one liter (1L) normal saline (NS), and was discharged home, feeling improved. Once home, the patient had recurrent vomiting, prompting presentation to the ED of the local academic medical center. Physical examination during her ED visit was unremarkable, including heart rate (HR) 54, blood pressure (BP) 124/65, respiratory rate (RR) 16, and temperature (T) 97.7°F. Diagnostic evaluation in the ED included basic labs (complete blood count [CBC], basic metabolic panel [BMP], and urinalysis [UA]). All labs were within normal for hospital reference ranges, except for 2–5 RBC/hpf (red blood cell per high power field) and trace ketones on urinalysis. The patient received 4mg IV ondansetron, 0.625mg IV droperidol, 1L NS (normal saline) IV bolus, and was discharged home feeling improved.

Patient returned to the ED 4 days later with ongoing nausea, resulting in poor oral intake, and generalized weakness. She noted frequent falls from standing and difficulty walking because of “weakness.” Initial vital signs were as follows: BP 79/53, HR 93, RR 18, and T 98.5°F. Subsequent blood pressures during her ED stay ranged from 124/63 to 148/82. Physical exam revealed a thin woman with dry mucous membranes, clear lungs, normal heart sounds, and a benign abdominal exam. Notably, neurologic exam revealed equal and intact strength in all extremities. BMP, CBC, and UA were repeated. All labs were again within hospital reference range except for potassium 3.0mmol/L (RR 3.5–4.8mmol/L), and urinalysis with 6–10 RBCs/hpf and large ketones. She was again treated with 8mg IV ondansetron, 0.625mg IV droperidol, 40mg IV pantoprazole, and 2L NS IV bolus, with resolution of her symptoms, and was discharged home with a prescription for potassium chloride tablets.

The patient returned to the same ED two days later, reporting that her nausea and vomiting had resolved and that she was now tolerating a regular diet, but had continued to have falls and over the prior hour had developed slurred speech and bilateral hand weakness. These symptoms, however, had resolved prior to ED arrival. Initial vital signs for this third ED visit were as follows: BP 102/63, HR 73, RR 18, and T 99.1°F. Patient’s physical examination again revealed dry mucous membranes, normal heart and lung sounds, and benign abdominal exam. Her neurologic exam at this time was documented as intact muscle tone, normal cranial nerve exam, and intact coordination. Laboratory testing included BMP, magnesium level, phosphate level, ionized calcium level, troponin, CBC, vitamin B12 level, and urinalysis. All labs were within hospital reference range, except ionized calcium of 4.6mg/dL (RR 4.9–5.6mg/dL), Vitamin B12 level >4000pg/ml, (RR 210–911pg/ml), large ketones and 2–5 RBCs/hpf on urinalysis, and potassium 2.6mmol/L (RR 3.5–4.8mmol/L). The hypokalemia was supplemented with 20mg IV potassium chloride. Given neurologic symptoms and reported falls, patient underwent non-contrast computed tomography (CT) of her head, which demonstrated no traumatic intracranial hemorrhage or other pathology. The patient was admitted to the hospital for further evaluation of possible transient ischemic attack (TIA) and ongoing treatment of hypokalemia.

**Figure 1:**
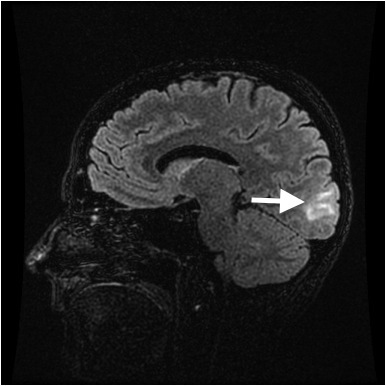
T2 weighted magnetic resonance imaging of Patient 1, showing hyperintensity within the occipital lobe.

The next day, MRI/MRA (magnetic resonance angiogram) head and neck with and without contrast showed symmetric T2 signal intensity within the occipital lobes, without evidence of infarction or enhancement, most consistent with PRES (Figure 1). Repeat neurologic exam by neurology service revealed bilateral left lower quadrantanopsia. Patient’s inpatient stay included extensive evaluation for autoimmune diseases, including a celiac panel, antinuclear antibodies, erythrocyte sedimentation rate, C-reactive protein, and anti-SCL-70 antibodies. All of these laboratory tests were resulted as with normal hospital reference ranges. Patient declined a lumbar puncture for further laboratory examination of her cerebral spinal fluid.

The patient had steady improvement of her symptoms over a five-day hospital course and was discharged with outpatient physical, occupational, and speech therapies. The patient underwent outpatient MRI three months after hospital discharge, which showed complete resolution of prior PRES findings. Her visual field deficits resolved, and she has returned to work.

### Case 2

A 69-year-old woman with past medical history of end-stage renal disease (ESRD) on renal dialysis, poorly controlled type-II diabetes mellitus, and hypertension, presented to a regional community ED with complaints by husband of altered mental status and rash. Specifically, husband noted that since being discharged from a community hospital one week prior, after a two-day stay for pneumonia, the patient had become increasingly fatigued and weak. During the patient’s hospitalization for pneumonia, her blood pressure medications (including carvedilol, clonidine, doxazosin, and lisinopril) had all been stopped due to mild hypotension. A targetoid rash had also formed on her arms, left leg, posterior shoulders, and neck. Her weakness had progressed until morning of her ED presentation, when she became acutely confused. She had called out for her husband and was complaining of pain in her bilateral shoulders and arms but was unable to further describe her discomfort.

Per report by outside hospital, patient’s initial vital signs were notable for a blood pressure of 200/110, HR 72, and normal temperature. She was given 2.5mg IV enalaprilat, which improved her BP from 200/110 to 185/88. While undergoing a non-contrast head CT at the outside hospital, patient had a two to three minute long generalized tonic-clonic seizure, for which she received 2mg IV lorazepam. Head CT was interpreted as normal. Request for transfer was then made to the ED of the local academic medical center.

On arrival, the patient’s vital signs included the following: BP 197/59, HR 80, RR 20, and T 99.7°F. Her physical exam revealed an ill-appearing woman with small yet reactive pupils, supple neck, normal heart and lung sounds, and benign abdominal exam. Her neurologic exam revealed a lethargic patient, only oriented to self, who could not hold up her extremities to gravity. Dermatologic exam revealed diffuse erythematous rash on bilateral upper and lower extremities and upper neck. Laboratory examination included the following: arterial blood gas, comprehensive metabolic panel (CMP), CBC, and urinalysis. All were within normal limits of hospital reference ranges except for the following: white blood cell count (WBC) 11.6 (RR 3.8–10.5 k/uL), platelets 41 (RR 160–370 k/uL), and creatinine (Cr) 5.48 (RR 0.55–1.05 mg/dL). Patient’s WBC was elevated compared to baseline, her platelet count was low compared to baseline. Since she was two days short of her next dialysis, her Cr level was at baseline. The neurology service was consulted from the ED. Given concern about the rash and possible low-grade temperature, empiric broad-spectrum antibiotics were started (1g vancomycin, 3g meropenem, and 600mg acyclovir).

**Figure 2:**
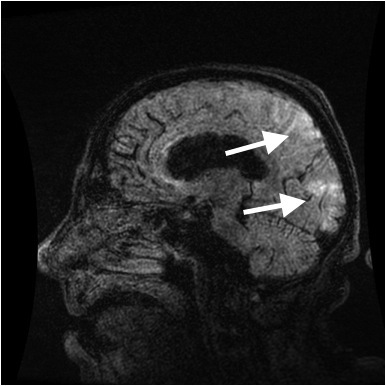
T2 weighted magnetic resonance imaging of Patient 2, with hyperintensity of posterior parietal and occipital lobes.

The patient was admitted to the inpatient medicine service with a working differential diagnosis of thrombotic thrombocytopenic purpura (TTP), encephalitis or meningitis, and hypertensive emergency. MRI of the head without contrast was obtained which showed T2 and fluid-attenuated inversion recovery (FLAIR) signal hyperintensity located in a symmetric fashion within the bilateral occipital and posterior parietal lobes, interpreted as characteristic of PRES (Figure 2).

The patient’s inpatient stay focused on blood pressure management and evaluation of altered mental status. A lumbar puncture was performed which showed only one nucleated cell. Cultures of blood, spinal fluid, urine, and sputum were all negative. Dermatology was consulted regarding the patient’s rash and KOH testing was suggestive of tinea corporis.

The patient continued to have a fluctuating mental status. An electroencephalogram (EEG) showed a non-epileptiform pattern with generalized nonfocal slowing consistent with a global impairment. On hospital day 7, patient was made comfort care measures only by family and died shortly thereafter.

On autopsy, the brain was grossly and microscopically normal. Systemic atherosclerotic disease was noted, as well as coronary artery disease, and chronic obstructive pulmonary disease.

### Case 3

A 46 year old woman with a history of thyroid cancer (status post thyroidectomy) and adenoid cystic carcinoma (status post resection and radiation) presented to the ED of a local academic medical center complaining of 24 hours of a left-sided, throbbing headache, associated with nausea, vomiting, and diaphoresis. She stated that the headache came on rapidly over five seconds. Physical exam included BP 152/103, HR 89, RR 17, and Temp 98.6°F. Patient had a non-focal neurologic exam, including intact cranial nerves, strength, sensation, and coordination. CT and CT angiogram of her head were obtained, and showed no evidence of intracranial hemorrhage, vascular abnormality (e.g. aneurysmal dilatation or ateriovenous malformation), or any other acute abnormalities. The patient was strongly encouraged to undergo further diagnostic testing with a lumbar puncture for evaluation of a subarachnoid hemorrhage, but declined. After treatment with 1.25mg IV droperidol, 25mg IV diphenhydramine, and 0.5mg IV hydromorphone, the patient’s pain was improved and she requested discharge. She left the ED at 2:15 AM.

**Figure 3:**
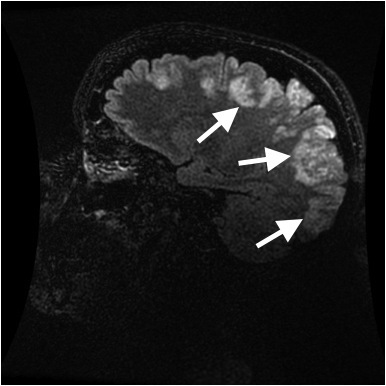
Initial T2 weighted magnetic resonance imaging of Patient 3 on second emergency department visit, showing increased T2 intensity throughout the cortical and subcortical gray matter of the bilateral frontal, parietal, occipital and posterior temporal lobes.

The patient returned via ambulance 17 hours later, at 7:00 PM in the evening, after having a witnessed three-minute generalized tonic-clonic seizure at home, and another one-minute generalized tonic-clonic seizure during transport. The latter resolved with 5mg intranasal midazolam. She arrived to the ED alert, oriented to self and location, but not time. She complained of a severe generalized headache. Initial vital signs were as follows: BP 183/113, HR 143, and RR 13. Finger stick glucose was 195mg/dl (ranger 70–99mg/dL). Laboratory testing included CMP, magnesium level, phosphate level, CBC, thyroid stimulating hormone, alcohol, urine drug screen, and urinalysis which were all normal except for positive opiates and benzodiazepines on urine drug screen (thought most likely secondary to medications given to patient during her ED stay earlier that day), WBC count 12.8, phosphate 1.9mg/dL[RR 2.5–4.5mg/dL] and potassium 3.1mg/dL. Head CT was repeated and showed Ill-defined areas of low-attenuation in the cortical and subcortical regions of the bilateral posterior parietal and occipital lobes. The patient’s neurologic exam was remarkable only for a new left inferior homonymous quadrantanopsia. She was loaded with 1.5g levetiracetam and admitted to the neurology service. MRI/MRA of the head and neck with and without contrast subsequently demonstrated abnormal increased T2 and T2 FLAIR signal intensity throughout the cortical and subcortical gray matter of the bilateral frontal, parietal, occipital and posterior temporal lobes, with scattered areas of restricted diffusion within the posterior cortex of the bilateral parietal regions, concerning for PRES (Figure 3). The patient underwent lumbar puncture, with normal cerebrospinal fluid studies.

**Figure 4:**
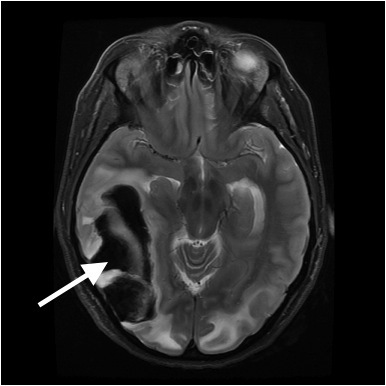
T2 weighted magnetic resonance imaging of Patient 3 on hospital day 4 with massive right-sided temporo-occipital intraparenchymal hemorrhage.

Once admitted, her chest x-ray showed patchy opacities bilaterally, and her oxygen saturation on repeat measures slowly dropped to 80%. As such, patient was intubated on her first hospital day for presumed aspiration pneumonia. She was treated with ampicillin and sulbactam, and her hypoxemia steadily resolved. She received daily enoxaparin injections for thrombosis prophylaxis. On hospital day four, the patient was noted to have a sudden decline in mental status and repeat MRI showed a massive right-sided temporoccipital intraparenchymal hemorrhage (Figure 4). The patient was taken for decompressive craniectomy and drainage.

Her mental status slowly improved and she was transferred to the rehabilitation service on hospital day 21. Her subsequent course was complicated by a surgical wound infection, requiring surgical washout. She was discharged home with ongoing physical, occupational, and speech therapies 41 days after her initial presentation. Two months after discharge, the patient has some mild to moderate persistent short-term memory loss and persistent left visual field deficit, but is otherwise neurologically intact.

## Discussion

These three cases demonstrate the extremely variable presentation and clinical course of patients found to have PRES on neuroimaging. The medical literature in general, and emergency medicine (EM) literature in particular, is very limited with respect to prevalence, epidemiology, etiology, and management of PRES. In the few published reviews of patients diagnosed with PRES, however, the most common clinical manifestations were seizure (74%–91.7% of patients), followed by encephalopathy (28%–92%), headaches (26%–83.3%) and visual disturbances (20%–62.5%).^5^–^7^ Hypertension was the most common associated co-morbid condition (53%–91.7%), followed by kidney disease (20.8%–45%), autoimmune disease (45%), malignancy (32%), organ transplant (24%), cytotoxic medications (19%), renal artery stenosis (12.5%), sepsis (7%), pre/eclampsia (6%), Takayasu’s arteritis (4.2%), Sheehan syndrome (4.2%) and multi-organ dysfunction (1%) for the patients included in these reports.^5^–^7^ PRES has now also been well described in the pediatric population.^8^ Given the wide and variable range of patient presentations, PRES is a difficult diagnosis to make in the ED.

Several pathophysiologic mechanisms have been thought to lead to the development of PRES. The current theory is that endothelial dysfunction leads to edema of the surrounding tissues.^1^ The posterior cerebrum is thought to be especially sensitive to such injuries because of poor sympathetic innervation of the vasculature.^9^ This could explain why PRES can present as a complication of a wide array of disease processes which all can lead to endothelial dysfunction.

Two studies in the literature report their experience in treating patients with PRES. Prompt lowering of blood pressure, treatment of associated seizures and removal of the causative agent are recommended in the management of PRES.^10^,^11^ A mean arterial blood pressure reduction to 105–125mmHg is suggested with no more than 25% of this reduction occurring in the first hour. First line agents to achieve this effect are calcium channel blockers (e.g. nicardipine) or beta-blockers (e.g. labetalol). Second line agents to consider are sodium nitroprussiate and hydralazine. Nitroglycerin should be specifically avoided secondary to reports of worsening cerebral edema likely mediated by enhancing cerebral vasodilation.^10^,^11^

Management of seizures has been recommended to follow that of other epileptic seizures. This includes benzodiazepines, such as lorazepam or diazepam, as first line agents. Second line agents include fosphenytoin or phenobarbital. In pregnant patients one can consider magnesium sulfate. Refractory seizures can be managed with propofol or pentobarbital. There are reports of patients treated with valproic acid for seizures.^10^,^11^

A few key points should be highlighted with respect to the above three cases. The patient in Case 1 had only mild hypertension (140s/80s), although this was significantly higher than the patient’s baseline blood pressures, which were systolic blood pressures in the 80s–90s range. Her history of esophageal dysmotility is also suggestive of a possible autoimmune process, such as scleroderma. However, her subsequent rheumatologic workup was negative. Of note, the patient subsequently submitted multiple complaints to regulatory bodies. A thorough review and discussion of the case during EM monthly Morbidity and Morality Conference case conference revealed only 12 out of 23 attending emergency physicians had ever heard of PRES, and only five had cared for a PRES patient in the past.

The patient in Case 2 presented with marked hypertension, altered mental status, and seizures as is typical of PRES presentations. It is atypical, however, for PRES to proceed to death. Autopsy did not reveal any alternate cause of death. The patient in Case 3 had a subsequent intraparenchymal hemorrhage. The exact etiology of the bleed in this case is not clear, although it may have been secondary to prolonged hypertension. PRES itself has been associated with intracranial hemorrhage, as well. One retrospective series of 263 patients with PRES found an intracranial hemorrhage prevalence of 19.4%, of which 90% were intraparenchymal hemorrhages.^12^ The majority of these (63%) were small punctate bleeds, with only 8.7% resulting in extensive hemorrhages, as seen in case 3. All intraparenchymal bleeds were located within or near the area of initial PRES-related parenchymal edema.

These cases highlight the difficulty in diagnosing PRES in the emergency department. Patient 1 and Patient 3 both had multiple ED visits prior to being admitted and diagnosed with PRES. Any patient who presents with altered mental status, headache, or seizure may benefit from consideration of PRES in the differential diagnosis. Such a consideration should prompt earlier MRI and neurology consultations. This is especially important because earlier diagnosis will lead to a more proactive search for, and treatment of, underlying causes. Of note, there are ongoing research studies looking into possible laboratory markers of PRES, including lactate dehygrogenase.^13^ While such markers, at present, will not be specific, a sensitive laboratory study can also assist evaluation and management of such patients.

## CONCLUSION

PRES can present to the ED in a variety of ways, and is associated with a wide variety of underlying pathology, as illustrated by these three cases. With the increasing awareness of this diagnosis by emergency physicians and increasing use of MRI in the ED, a PRES diagnosis may be made more frequently in the ED in the future. Ideally, earlier identification of PRES will result in more expedited evaluation and treatment, and better short and long-term outcomes for ED patients.

## References

[1] Hinchey J, Chaves C, Appingnani B (1996). A Reversible Posterior Leukoencephalopathy Syndrome. N Eng J Med.

[2] Hobson EV, Craven I, Blank SC (2012). Posterior reversible Encephalopathy Syndrome: A truly treatable neurologic illness. Perit Dial Int.

[3] Lee VH, Temes RE, John S (2013). Posterior Reversible Leukoencephalopathy Syndrome with Global Cerebral Edema and Herniation. Neurocrit Care.

[4] Golombeck SK, Wessig C, Monoranu CM (2013). Fatal atypical reversible posterior leukoencephalopathy syndrome: a case report. J Med Case Rep.

[5] Fugate JE, Claassen DO, Cloft HJ (2010). Posterior reversible encephalopathy syndrome: associated clinical and radiologic findings. Mayo Clin Proc.

[6] Lee VH, Wijdicks EF, Manno EM, Rabinstein AA (2008). Clinical spectrum of reversible posterior leukoencephalopathy syndrome. Arch Neurol.

[7] Ni J, Zhou LX, Hao HL (2011). The clinical and radiological spectrum of posterior reversible encephalopathy syndrome: a retrospective series of 24 patients. J Neuroimaging.

[8] Endo A, Fuchigami T, Hasegawa M (2012). Posterior Reversible Encephalopathy Syndrome in Childhood. Pediatr Emer Care.

[9] Schwartz RB, Jones KM, Kalina P (1992). Hypertensive encephalopathy: findings on CT, MR imaging, and SPECT imaging in 14 cases. AJR Am J Roentgenol.

[10] Servillo G, Bifulco F, De Robertis E (2007). Posterior reversible encephalopathy syndrome in intensive care medicine. Med Hypotheses.

[11] Striano P, Striano S, Tortora F (2005). Clinical spectrum and critical care management of Posterior Reversible Encephalopathy Syndrome (PRES). Med Sci Monit.

[12] Sharma A, Whitesell RT, Moran KJ (2010). Imaging pattern of intracranial hemorrhage in the setting of posterior reversible encephalopathy syndrome. Neuroradiology.

[13] Vargas M, Servillo G, Striano P (2012). Serum lactate dehydrogenase as early marker of posterior reversible encephalopathy syndrome: keep your eyes open. Anesth and Intensive Care.

